# Predicting the Risk of Hypertension Based on Several Easy-to-Collect Risk Factors: A Machine Learning Method

**DOI:** 10.3389/fpubh.2021.619429

**Published:** 2021-09-24

**Authors:** Huanhuan Zhao, Xiaoyu Zhang, Yang Xu, Lisheng Gao, Zuchang Ma, Yining Sun, Weimin Wang

**Affiliations:** ^1^Institute of Intelligent Machines, Hefei Institutes of Physical Science, Chinese Academy of Sciences, Hefei, China; ^2^Science Island Branch of Graduate School, University of Science and Technology of China, Hefei, China; ^3^School of Computer and Information Engineering, Chuzhou University, Chuzhou, China; ^4^Institute of Health Management, Chinese People's Liberation Army (PLA) General Hospital, Beijing, China

**Keywords:** hypertension, risk prediction, machine learning method, easy-to-collect, lifestyle

## Abstract

Hypertension is a widespread chronic disease. Risk prediction of hypertension is an intervention that contributes to the early prevention and management of hypertension. The implementation of such intervention requires an effective and easy-to-implement hypertension risk prediction model. This study evaluated and compared the performance of four machine learning algorithms on predicting the risk of hypertension based on easy-to-collect risk factors. A dataset of 29,700 samples collected through a physical examination was used for model training and testing. Firstly, we identified easy-to-collect risk factors of hypertension, through univariate logistic regression analysis. Then, based on the selected features, 10-fold cross-validation was utilized to optimize four models, random forest (RF), CatBoost, MLP neural network and logistic regression (LR), to find the best hyper-parameters on the training set. Finally, the performance of models was evaluated by AUC, accuracy, sensitivity and specificity on the test set. The experimental results showed that the RF model outperformed the other three models, and achieved an AUC of 0.92, an accuracy of 0.82, a sensitivity of 0.83 and a specificity of 0.81. In addition, Body Mass Index (BMI), age, family history and waist circumference (WC) are the four primary risk factors of hypertension. These findings reveal that it is feasible to use machine learning algorithms, especially RF, to predict hypertension risk without clinical or genetic data. The technique can provide a non-invasive and economical way for the prevention and management of hypertension in a large population.

## Introduction

The expert system can learn medical knowledge and expert experience, and finally simulate expert diagnosis and treatment ideas and draw conclusions, which can help diagnose and analyze human diseases (1). The management of human diseases urgently needs an expert system to assist in real-time diagnosis and personalized prevention or treatment guidance. With the development of artificial intelligence (AI) and fuzzy logic, the effectiveness of expert systems in the medical field has been widely reported (2–6). As a core technology of AI, machine learning is the foundation of expert systems (7). Supervised machine learning algorithms have been used in traditional disease risk prediction models to improve the accuracy of classification (8).

Hypertension is a widespread cardiovascular disease (9, 10), which has been the first risk factor of death and the third risk factor of the economic burden (11). Moreover, most of the occurrence and development of hypertension are symptomless. The continuous rise of blood pressure in hypertension patients usually has complications, such as arteriosclerosis, myocardial infarction, and stroke (12, 13). Luckily, previous studies consistently indicated that the early stage of lifestyle modification can prevent and control the development of hypertension (14). Therefore, it is critical to access the individuals' risk of hypertension and to screen hypertension early. The hypertension risk prediction model can identify high-risk groups and screen out hypertension patients at an early stage. Individuals can cease the unhealthy behaviors to prevent and control the management of hypertension, with the early warning from lifestyle risk factor indicators (15–18). Therefore, identifying lifestyle risk factors of hypertension and early identification of hypertension play an important role in the prevention and management of hypertension.

Existing hypertension risk prediction approaches can be roughly classified into queue-based and cross-sectional data-based. The former focuses on obtaining the absolute risk of hypertension and requires long-time longitudinal data, which limits the application of modeling methods. In contrast, the latter one employs features extracted from cross-sectional data to evaluate the current risk of hypertension and screen out hypertension, which is also of great value in the prevention and management of hypertension. Probably the most related investigation in hypertension has recently been focusing on the current risk prediction by analyzing clinical indicators or genetic information. Ture et al. (19) constructed different hypertension prediction models based on clinical indicators. The performance of neural networks is superior to decision trees and traditional statistical algorithms. Elizabeth Held (20) generated a hypertension prediction model by using LR based on age, sex, smoke, age^*^sex and genetic information. With the help of the K-means algorithm to avoid sample imbalance to obtain balanced experimental data, Wang et al. (21) utilized a neural network to establish a hypertension prediction model. A Swedish hypertension risk model (22) employed LR to study heart rate, memory and metabolic characteristics and their association with the prevalence of hypertension. In a multi-ethnic study, Lopez-Martinez F et al. (23) utilized LR to build a hypertension prediction model based on the classification values of each risk factor, and the performance of the model was better than random guessing.

Extensive research efforts have been dedicated to the issue of hypertension risk prediction. However, there are still great difficulties in applying these models in practical applications in a large population because of the complexity of predictors' collection and the unsatisfied predictive ability of these models. Firstly, the predictors of these current models all contain biochemical indicators or genetic information, which requires a complex measurement and cannot be achieved in some situations, such as rural areas or some community health service centers. Secondly, compared with models based on biochemical indicators or genetic information, the model based on lifestyle risk factors can effectively identify the risk level of hypertension and contribute to targeted intervention. Thirdly, It is urgent to develop an effective model using only easy-to-collect risk factors (no biochemical and genetic information) to improve predictive performance. Furthermore, the poor interpretability of previous prediction models limits their practical application.

The objectives of this study were to evaluate and compare the performance of four different machine learning algorithms in predicting the risk of developing hypertension from easy-to-collect information. And choose the best machine learning algorithm to develop a risk prediction model of hypertension based on easy-to-collect information. The four machine learning algorithms used in this study were RF, CatBoost, MLP neural network and LR.

## Materials and Methods

### Material

The data set used to construct the model in this paper comes from a physical examination center of a hospital in Beijing in China. A total of 29,750 cases of complete data were collected. Among them, there are 10,650 cases of hypertension and 19,100 cases of normal. Most of the normal cases are between 18 and 70 years old, and most hypertension cases are between 20 and 75 years old. To ensure a similar age distribution between hypertension and normal cases, the age of samples is further restricted between 20 and 70 years old. For the selected 10,625 hypertension cases and 19,080 normal controls, we took the following measures to clean up the data: eliminate the subjects with significant outliers (more than or equal to 3 times four Quantile intervals). After screening and data cleaning of inclusion and exclusion criteria, 10,623 cases of hypertension and 19,077 cases of normal controls were finally included in this study. We promise to keep the patients' information strictly confidential. According to the Helsinki Declaration, the study was approved by the Ethics Committee of the Hefei Institute of Physical Science, Chinese Academy of Sciences (No. Y-2018-29).

The anthropometric information and blood pressure of the subjects were measured by professional medical workers using standard measurement methods. According to the diagnostic criteria of the “Chinese Hypertension Prevention Guide,” hypertension is defined as having been diagnosed as hypertension in the hospital or the average systolic blood pressure> = 140 mmHg or diastolic blood pressure> = 90 mmHg in this physical examination. The anthropometric indicators mainly include height, weight and WC. During the measurement, the subjects were required to wear light clothes and maintain a correct standing posture. BMI is calculated according to the standard formula [BMI = weight/height^2^ (kg/m^2^)]. WC is measured by using the tape around the subject for one circle at 1 cm above the navel (24). Professional medical staff used standardized epidemiological questionnaires to complete interviews of subjects for collecting basic demographic and lifestyle information. Family history refers to the hypertension status of one's parents. Smoke is defined as smoking every day and has been smoking for more than 6 months (25). Drink refers to drinking at least once a week and has been drinking for more than 6 months (26). Occupation refers to one's occupation type. The physical activity presents physical activity status, frequent physical activity refers to 30 min of moderate-intensity exercise performed at least three times a week (27). A healthy diet is defined as the total score of the healthy eating index (HEI) >51 (28). Psychological pressure refers to the total score of the Perceived Stress Scale (PSS) > = 43 (29). Table 1 shows the details of these variables.

**Table 1:** Variable description information.

**Variables**	**Type**	**Description**	**Mean**
Age	Continuous	Age at health checkup (years)	Age
BMI	Continuous	BMI at health checkup (kg/m^2^)	BMI
WC	Continuous	WC at health checkup (cm)	WC
Gender	Categorical	0: men 1: women	Men Women
Family history	Categorical	0: No family history of hypertension 1: Only one of the parents has hypertension 2: Both parents have hypertension	No Parent Parents
Occupation	Categorical	0: Civil servants 1: Other occupation	Civil servants Other occupations
Smoke	Categorical	0: not has regular smoking habits 1: has regular smoking habits	No yes
Drink	Categorical	0: not has regular drinking habits 1: has regular drinking habits	No yes
Health diet	Categorical	0: unhealthy diet 1: healthy diet	No yes
Physical activity	Categorical	0: not frequent physical activity 1: frequent physical activity	No yes
Psychological pressure	Categorical	0: has no pressure at all or occasional small pressure 1: has high pressure	No yes
Hypertension	Categorical	0: non-hypertension 1: hypertension	No yes

*BMI, Body Mass Index; WC, waist circuit*.

To verify the performance of the four machine learning algorithms on our data, we randomly divided the dataset into a training set and a test set according to ratio 4:1. There is no significant difference in each variable between the training set and the test set. The main characteristics of the training set and the test set are shown in Table 2.

**Table 2:** The main characteristics of the training set and test set.

**Feature**	**Training set**		**Test set**	
	**Hypertension (*n* = 8,492)**	**Normal (*n* = 15,268)**	**Hypertension (*n* = 2,131)**	**Normal (*n* = 3,809)**
Age (years)	48 (44–54)	46 (41–50)	49 (45–54)	46 (41–50)
BMI (kg/m^2^)	26.9 ± 3.1	24.7 ± 3.2	26.9 ± 3.1	24.7 ± 3.2
WC (cm)	92.9 ± 9.4	86.4 ± 10.4	93.2 ± 9.2	86.4 ± 10.9
occupation (civil servants)	53.9%	45.7%	55.1%	45.9%
gender (male)	82.2%	64.4%	82.4%	64.2%
**Family history (%)**				
parents	25.1%	12.9%	24.3%	13.3%
parent	37.6%	27.6%	37%	27.4%
Smoke (%)	51.1%	38.2%	49.9%	38.7%
Drink (%)	79.2%	66.1%	78.4%	66.7%
Healthy diet (%)	58.7%	65.5%	59.6%	66.3%
Physical activity (%)	33.1%	36.5%	32.2%	34.9%
Psychological pressure (%)	37.6%	36.5%	36.2%	36.3%

*Continuous variables are expressed as median (interquartile range), mean (standard deviation), and categorical variables are expressed as frequency (%)*.

### Feature Selection

The variables used to construct hypertension risk prediction model must meet the following two conditions: (1) it is an easy-to-collect variable, including basic demographic information, anthropometric information, or lifestyle information; (2) It is a variable statistically significant to hypertension in univariate logistic regression analysis (*p* < 0.05) (30).

### Machine Learning Algorithms

In this study, we used four machine learning techniques to develop four models based on easy-to-collect variables to predict the risk of hypertension: RF, CatBoost, MLP neural network and LR.

#### RF

RF is an ensemble machine learning method with decision trees as the base classifier (31). Each decision tree is built based on various sub-datasets and features. Therefore, each decision tree is different and independent, and finally, the classification result from the voting results of multiple decision trees is obtained. This approach allows reducing variance in decision trees (32). Thus, RF can analyze the classification characteristics with complex interactions, and it is very robust to noisy data and data with missing values. Meanwhile, the learning speed of RF is also very fast.

#### CatBoost

CatBoost is a new ensemble algorithm based on decision tree gradient boosting (33). CatBoost uses combined categorical features, which can take advantage of the connections between features and greatly enrich the feature dimension. Therefore, CatBoost is intrinsically more efficient and has better predictive performance compared with the traditional boosting algorithm in the case of categorical features.

#### MLP Neural Network

As a non-linear mapping model, MLP neural network is flexible and effective in modeling complex relationships between inputs and outputs (34). It includes an input layer, a hidden layer, and an output layer. Each layer is fully connected to the previous layer. The MLP neural network is trained according to the error backpropagation algorithm (35). It performs error analysis on the training and expected results each time, which helps change the weights and thresholds to obtain a model that the outputs are consistent with the expected results step by step. The process can be terminated when the error rate reaches sufficiently small.

#### LR

LR is a generalized linear regression analysis algorithm that can explore the relationship between a categorical dependent variable and several independent variables (30) and connect the values of the independent variables with the probability of the event defined by the dependent variable. The LR algorithm assumes that the predicted value is the linear addition of all products of independent variables and corresponding coefficients.

### Hyper-Parameters Tuning and Model Development

To evaluate the performance of four machine learning models, we randomly divided the data into a training set and a test set according to ratio 4:1. For the four machine learning techniques, the training set was used to adjust the model parameters and construct the model, and the test set was used to evaluate the performance of the model. The training set was divided into a training subset and a verification set according to ratio 9:1, AUC of 10-fold cross-validation was used as the evaluation indicator to adjust the model parameters for constructing the optimal model. The training set was used to fit the model and generate the final model after the optimal parameters were determined. All the algorithms were implemented in Python 2.7.

### Evaluation Metrics

The performance of the predictive model is evaluated by ROC (Receiver Operating Curve) curve, accuracy, sensitivity, specificity, and Youden index. Accuracy refers to the ratio of correctly classified samples to the total number of samples. Sensitivity refers to the proportion of positive samples that are predicted to be positive. Specificity refers to the proportion of negative samples that are predicted to be negative. The classification confusion matrix (36) is shown in Table 3.

**Table 3:** Classification confusion matrix.

**Real situation/ Predicted value**	**Hypertension**	**Normal**
Hypertension	TP	FN
Normal	FP	TN

Among them, TP is the number of positive samples judged as positive, FN is the number of positive samples judged as negative, FP is the number of negative samples judged as positive, and TN is the number of negative samples judged as negative.


$$\begin{array}{l} Accuracy\;=\;\frac{TP+TN}{TP+TN+FP+FN}\\ Sensitivity\;=\;\frac{TP}{TP+FN}\\ Specificity\;=\;\frac{TN}{TN+FP}\\ Youden\;index\;=\;Sensitivity\;+\;Specificity\;-\;1 \end{array}$$

ROC curve is a curve drawn according to a series of different threshold values, with the true positive rate (sensitivity) as the ordinate and the false positive rate (1-specificity) as the abscissa. AUC (Area Under Curve) represents the area under the ROC curve. The value of AUC is equal to the probability that the prediction value is greater for a randomly given positive sample than a randomly given negative sample (37). The calculation formula of AUC is as follows:


$$\begin{array}{l} AUC=\int_{x=0}^{1}TPR\left({FPR}^{-1}\left(x\right)\right)dx \end{array}$$

In the above formula, TPR stands for true positive rate and FPR stands for false positive rate.

### Feature Importance

One weakness of machine learning methods is that the learning process is a black box operation, and the results are poorly interpretable. In this study, we calculated the importance of each feature to improve the interpretability of the model. To calculate the importance of a feature, we repeated the testing process 10 times. In each testing process, we successively permuted the values of each feature in the test set and calculated the corresponding decrease in the AUC. The importance of a feature is measured by the average decrease in the AUC of the test set. The larger the value means the greater the contribution of the feature to the model, that is the greater the importance of the feature.

## Results

### Selected Features

To select the input features for the prediction model, a univariate logistic regression analysis was utilized separately for 11 easy-to-collect hypertension risk factors on the training set. According to the variable inclusion criteria of statistical significance *p*-value < 0.05, psychological pressure was excluded (*p* = 0.097). Finally, 10 variables of age, gender, BMI, WC, family history, occupation, smoke, drink, healthy diet and physical activity were selected as the input features of the model. Table 4 presents the results of the univariate logistic regression analysis.

**Table 4:** Univariate logistical regression analysis for the presence of hypertension.

**Variables**	***B***	**OR (95% CI)**	***P-*value**
Age	0.061	1.063 (1.059–1.067)	<0.001
Gender	−0.935	0.392 (0.368–0.419)	<0.001
BMI	0.216	1.241 (1.230–1.253)	<0.001
WC	0.067	1.070 (1.067–1.073)	<0.001
Family history	0.602	1.825 (1.762–1.891)	<0.001
Occupation	−0.329	0.720 (0.683–0.759)	<0.001
Smoke	0.528	1.696 (1.607–1.789)	<0.001
Drink	0.667	1.948 (1.831–2.073)	<0.001
Healthy diet	0.290	1.336 (1.265–1.411)	<0.001
Physical activity	−0.153	0.858 (0.811–0.908)	<0.001
Psychological pressure	0.046	1.048 (0.992–1.107)	0.097

### Model Hyper-Parameters

Based on the 10 selected easy-to-collect risk factors, the training set was used to determine the optimal hyper-parameters for RF, CatBoost, MLP neural network and LR, respectively. The hyper-parameters of each model under optimal performance are shown in Table 5. Default values were set for other unlisted parameters in the four machine learning algorithms.

**Table 5:** Configuration of hyper-parameters in each machine-learning algorithm.

**Machine learning algorithm**	**Hyper-parameter name**	**Value range**	**Value**
Random forest	n_estimator	[3, 10, 30, 40, 50]	50
	max_features	[2, 4, 6, 8, 10, 11]	4
	bootstrap	[“true,” “false”]	false
	Random_state	[0, 1, 2]	0
CatBoost	depth	[2, 4, 6, 8, 10]	10
	iterations	[100, 300, 500, 600]	600
	Learning rate	[0.1, 0.2, 0.3]	0.3
MLP neural network	solver	[“lbfgs,” “sgd,” ”adam”]	adam
	activation	[“relu,” “identity,” “logistic,” “tanh”]	tanh
	hidden_layer_sizes	[(10), (20), (40), (60)]	(60)
	seed	[0, 1]	1

### Model Performance

**Figure 1:**
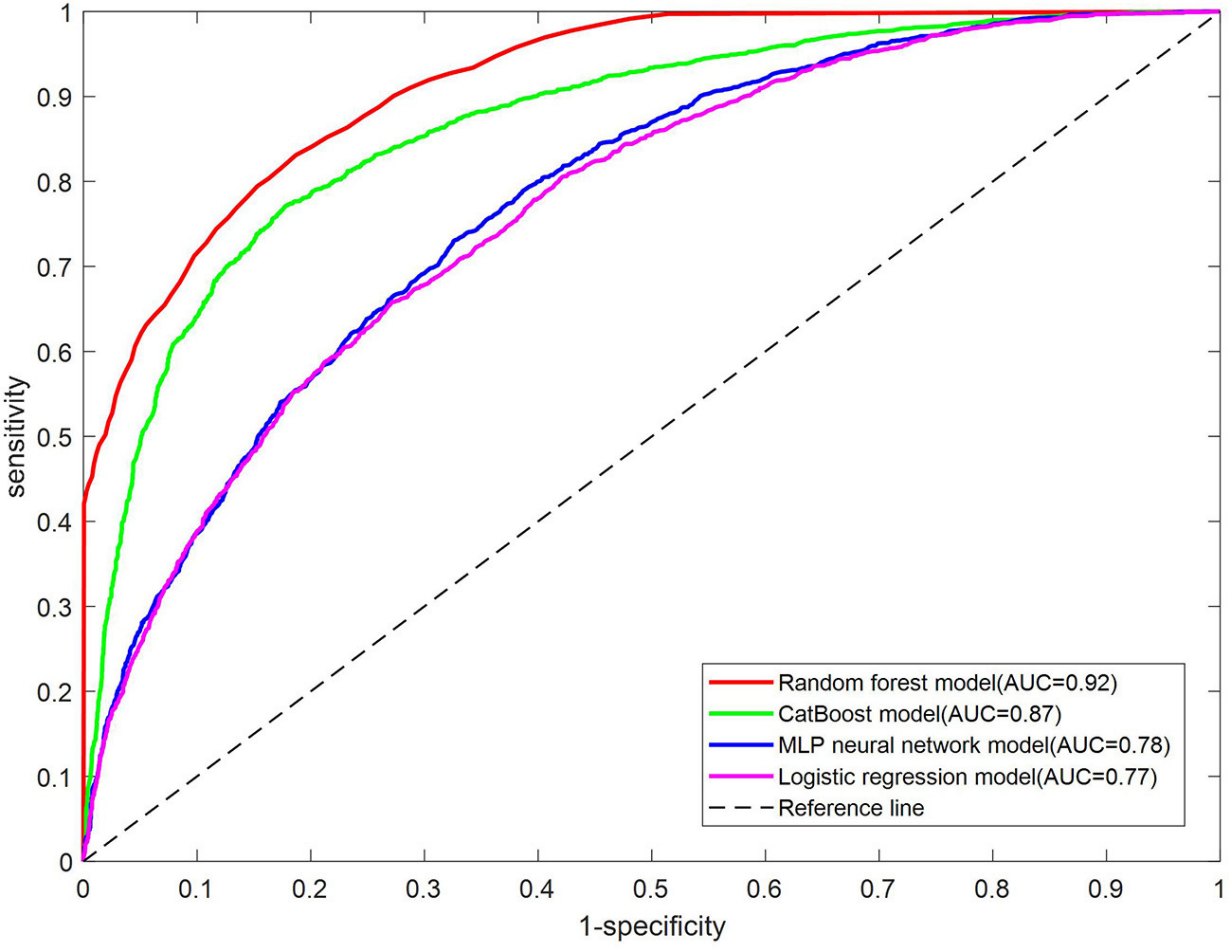
ROC curve for the test set of each model.

As shown in Table 6, the AUC for the test set on the RF model is the best, which is 0.92, followed by the CatBoost model with an AUC of 0.87, then the MLP neural network with an AUC of 0.78, and the LR model with an AUC of 0.77. The ROC curves on the test set of four models are shown in Figure 1. The RF model outperforms the other three models significantly.

**Table 6:** The performance of each model when the optimal threshold is selected.

**Model**	**AUC**	**Threshold**	**Accuracy**	**Sensitivity**	**Specificity**	**Yuden index**
RF	0.92	0.34	0.82	0.83	0.81	0.64
CatBoost	0.87	0.37	0.80	0.77	0.82	0.59
MLP Neural network	0.78	0.30	0.69	0.73	0.67	0.40
LR	0.77	0.39	0.70	0.66	0.73	0.39

**Figure 2:**
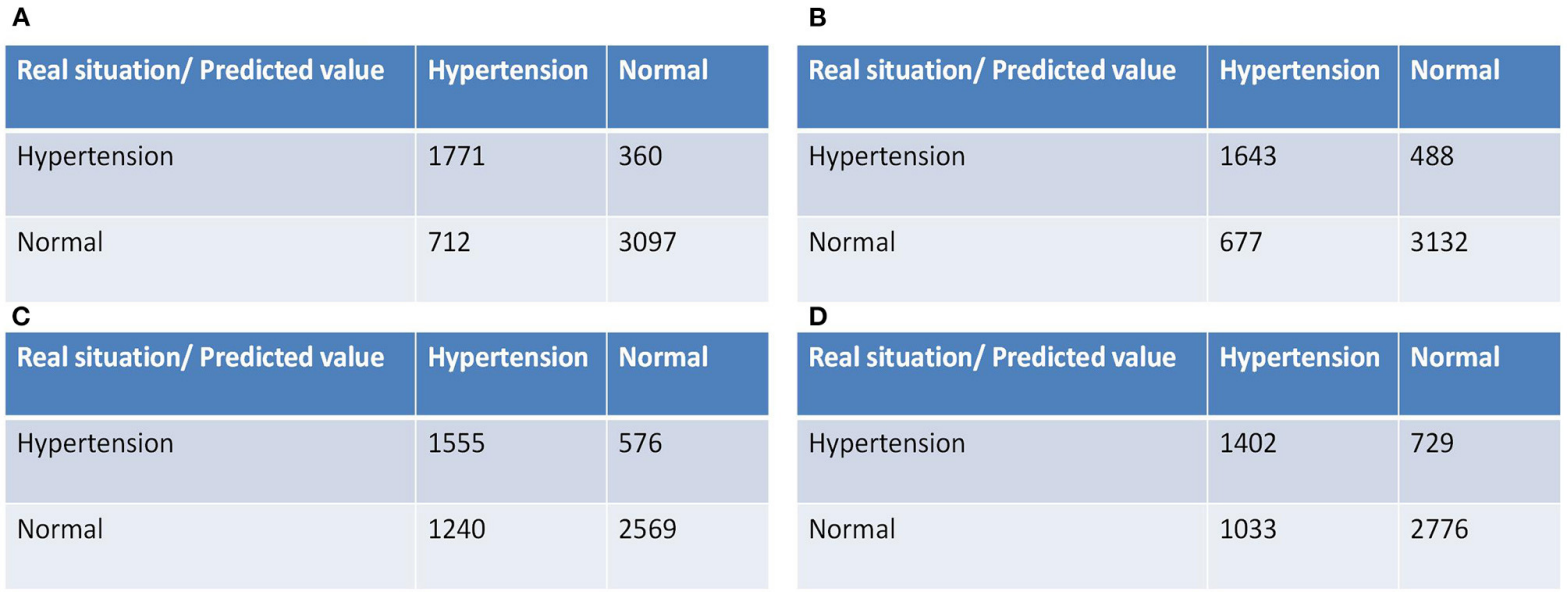
Confusion matrix for the test set of each model. **(A)** RF model, **(B)** CatBoost model, **(C)** MLP neural network model, **(D)** LR model.

Due to the imbalance phenomenon in the dataset, we adjusted the threshold to achieve the maximum Youden index. We obtained the classification confusion matrix after the threshold was determined for each model. Figure 2 shows the classification confusion matrix on the test set of four models. TP, FN, FP and TN for the RF model were 1771,360,712 and 3,097, respectively. The CatBoost model got a TP of 1,643, FN of 488, FP of 677, and TN of 3,132. For MLP neural network model, TP, FN, FP and TN were 1555, 576, 1,240 and 2,569, respectively. And the TP, FN, FP and TN were, respectively 1,402, 729, 1,033 and 2,776 for LR model. The RF model showed the best ability for identifying high-risk groups of hypertension compared with the other three models.

Refer to the accuracy, sensitivity, specificity and Youden index of the test set. As shown in Table 6, the best Youden index for the RF model is achieved (0.64) when the threshold is 0.34. At this time, the accuracy, sensitivity, and specificity for the model are 0.82, 0.83, and 0.81, respectively. The best Youden index for the CatBoost model is reached (0.59) when the threshold is 0.37. At this time, the accuracy, sensitivity, and specificity for the model are 0.81, 0.77 and 0.82, respectively. The best Youden index for the MLP neural network model is obtained (0.40) when the threshold is 0.30. At this time, the accuracy, sensitivity and specificity of the model are 0.69, 0.73 and 0.67, respectively. The best Youden index for the LR model is gotten (0.39) when the threshold is 0.39. At this time, the model got an accuracy of 0.70, a sensitivity of 0.66 and a specificity of 0.73, respectively. The results showed the performance of the RF model was still the best among the four models.

Based on the above results, the RF model performs the best on most evaluation metrics, including AUC, accuracy, sensitivity and Youden index, and achieves a favorable specificity, which was as good as the CatBoost model. Besides, RF was faster than CatBoost (2.24 s vs. 31.47 s). Therefore, RF had the overall best performance.

### Feature Importance

**Figure 3:**
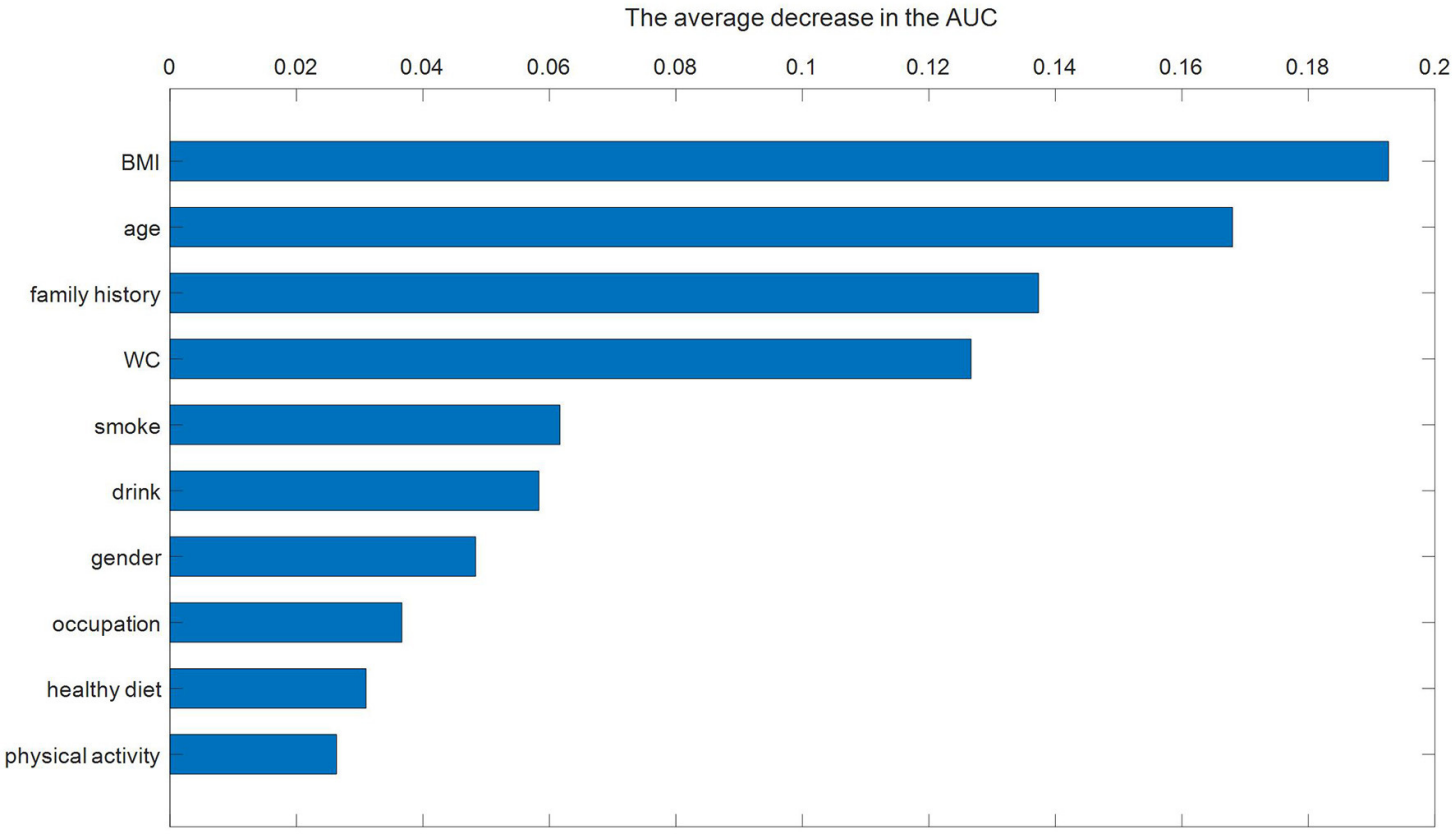
Feature importance ranking for the RF model.

We calculated the importance of each feature in the RF model, which achieved the best performance. The order of importance of each feature is shown in Figure 3. The top 4 features in order of importance were BMI, age, family history, and WC. Later, smoke, drink, gender, occupation, healthy diet, and physical activity were the features ranked 5 to 10 in order of importance.

## Discussion

### Principal Findings

In this study, four machine learning algorithms were evaluated and compared for hypertension risk prediction, based on easy-to-collect risk factors. The risk factors included 4 basic demographic indicators (gender, age, occupation and family history of hypertension), 2 anthropometric indexes (BMI and WC), and 4 lifestyle indicators (healthy diet, smoke, drink and physical activity). The results indicated that, compared with the LR model (AUC: 0.77), the performance of the three non-linear models was better. Thus, there is a non-linear relationship between the independent variables and the dependent variable. Among the three non-linear machine learning models, the RF model outperformed CatBoost and MLP neural network models and got an AUC of 0.92 and an accuracy of 0.82.

RF is a bagging ensemble algorithm based on multiple decision trees. The random selection of samples and features is further introduced in the training process. In the RF algorithm, there is no dependence between weak learners, and parallel operation can be achieved. All these attributes contribute to the excellent performance of RF in many classification studies (38, 39). CatBoost is an ensemble algorithm based on boosting, which is usually expert in dealing with categorical variables. As for the MLP neural network algorithm, it is more often utilized for processing unstructured data and data with complex structures. Thus, our sample data happens to meet the structured data and categorical features, which meet the demands of the RF algorithm and CatBoost algorithm. As expected, the two models have shown favorable performance in our data. In addition, the RF model outperforms CatBoost model (AUC: 0.92 vs. 0.87; accuracy: 0.82 vs. 0.80). Therefore, we believe RF is more reliable than CatBoost in terms of our data. Our results are consistent with a study of classification performance (38), in which the RF algorithm performed the best among the 179 classification algorithms on 121 UCI datasets.

Compared with statistical methods, the performance of the model built by the machine learning algorithm is better, but the disadvantage is the poor interpretability of the model. The process of machine learning to build a model is to learn the potential rules of input and output of training data, so it can fit complex non-linear relationships, and then get a trained model and predict new input data. However, the rule of the training data is unknown, so the process of machine learning is often called a black-box operation. To increase the transparency of the model and provide health education to residents in practical applications, we measure the effect of each feature on the performance of the RF model by calculating the average value of AUC reduction caused by permuting the values of each feature and explore the causal relationship between independent variables and dependent variable.

BMI, age, family history, and WC were the top four important features. Among them, BMI and age were the top two features. Wiewiora et al. (40) and Szpalski et al. (41) showed that obesity increased cardiac output and resistance of peripheral blood vessels, resulting in increased blood pressure. Among the many indicators of obesity, BMI was most closely related to hypertension (42). Mariunas et al. (43, 44) showed that with the increase of age, the elasticity of blood vessels became poor. To supply the blood demand of the whole body, blood pressure would rise. Therefore, age was an important risk factor for hypertension. The risk factors that followed were family history of hypertension and WC, A large population study (45) showed that people with a family history of hypertension had 1.79 times higher risk of hypertension than those without a family history of hypertension in China. The blood pressure level and prevalence of hypertension in those whose parents both had hypertension were significantly higher than those whose father or mother had hypertension. The prevalence of hypertension in those whose parents were both hypertension was about twice that of those without a family history of hypertension. These results are consistent with this study. WC was an important indicator of central obesity. Previous studies (46, 47) indicated that the risk of hypertension in centrally obese patients was much higher than that of the normal population. Therefore, WC was also an important risk factor for hypertension. Our results showed that the significance of WC on the incidence of hypertension was still great even after considering the effect of BMI on the incidence of hypertension. This was consistent with previous studies (42, 48). Smoke and drink were the next two important risk factors. Then, in order of importance, the risk factors were gender, occupation, healthy diet, and physical activity.

Although limited by the complexity of modern machine learning algorithms, we still cannot intuitively understand the relationship between independent and dependent variables in the model. However, the importance ranking of features indicated that the underlying rules in the data set learned by the RF algorithm were consistent with the findings of previous studies, which suggested that older and obese people had the highest risk of hypertension, and other unhealthy lifestyles would increase the risk of hypertension.

The RF model constructed in this study has made significant progress compared to similar previous hypertension prediction models. As shown in Table 7, we reviewed previous studies of hypertension prediction models. Ture et al. (19) built a hypertension prediction model based on lipoprotein (a), triglyceride, uric acid, total cholesterol and other biochemical indicators using the neural network, the calculated AUC of the model was 0.81. LR was used in the Swedish hypertension risk prediction model based on age, gender, BMI, heart rate, glycolipid parameters and other memory elements, the model got an AUC of 0.66 (22). Wang et al. (21) used a neural network to build a hypertension prediction model. The model with 10 hidden layers has the best performance with an AUC of 0.77. A hypertension prediction model based on genetic information, which was constructed using LR, achieved an accuracy of 0.77 (20). Lopez-Martinez et al. (23) utilized LR to construct a hypertension prediction model based on independent risk factors. The prediction model got an AUC of 0.73 and outperformed random guessing. Most models indicated a fair agreement with the final diagnosis for AUC values between 0.7 and 0.8. In this study, the RF model achieved a higher AUC (0.92) compared with the previous models. Studies have indicated that different ethnic populations have different characteristics of hypertension (49, 50), which likely impacts different AUCs for different models. Nevertheless, this study revealed a superior ability of the RF algorithm in distinguishing high-risk and low-risk populations of hypertension.

**Table 7:** Hypertension prediction models comparison.

**Author**	**Risk factors**	**N total**	**Type of model**	**AUC**
Ture et al. (19)	age, sex, family history, smoking habits, lipoprotein(a), triglyceride, uric acid, total cholesterol, BMI	694	Neural network	0.81
Fava et al. (22)	Age, sex, age^∧^2, age^*^sex, BMI, heart rate, Diabetes, alcohol, smoking, glycolipids, etc.	10,781	Logistic regression	0.66
Wang et al. (21)	Age,sex,marriage,education,income,height,weight,exercise,diabate, hyperlipemia, drink	30,871	Neural network	0.77
Held et al. (20)	Age, sex, smoke, age^*^sex, pedigree, single nucleotide polymorphism	637	Logistic regression	0.77
Lopez-Martinez et al. (23)	Age, Gender, ethnicity, BMI, smoking history, kidney disease, diabetes	19,799	logistic regression	0.73
Our research	Age, BMI, gender, WC, family history, occupation, smoke, drink, healthy diet, physical activity	29,700	Random forest	0.92

‘^*^’ refers to interaction term of two features.

On the other hand, the input variables of the previous hypertension prediction model all contained biochemical indicators or genetic information. Lipoprotein, triglyceride, uric acid, total cholesterol were required in Ture M's research (19). Although Wang et al. (21, 23) used a questionnaire to obtain predictors, the information about dyslipidemia and diabetes were required in Wang's research (21) and the information on kidney disease and diabetes were needed in Lopez-Martinez' s research (23). Genetic information was required when using Held E's model (20). Glucose and lipid parameters were the input variables of the model in Fava C's research (22). The acquisition of these variables requires biochemical testing, which makes these models unavailable for residents who cannot carry out biochemical testing on time. Thus, these models are not suitable and practical for hypertension prediction in a large population, which limits the application of these models in the prevention and management of hypertension. Different from previous prediction models constructed in other populations, the input variables of the RF model in this study are non-invasive and can be easily collected, which facilitates the application of the model.

### Limitations of This Study

This study still has several limitations. Firstly, the data set used for model construction in the study was derived from cross-sectional data of physical examination. Although the model cannot predict the absolute risk of hypertension, it can distinguish high-risk and low-risk groups of hypertension. Secondly, the data used in the study were collected from a local hospital, which means it can only represent the characteristics of hypertension among residents in this specific area. Therefore, the generalization of the RF model established in this study to other regions needs further research and confirmation. Lastly, we did not evaluate the effect of all possible lifestyle variables because they were not included in the health examination. Therefore, occupation was the only new risk factor for hypertension identified in this study. Further research needs to incorporate more lifestyle information.

## Conclusions

In this study, we evaluated and compared four machine learning algorithms in predicting hypertension risk based on easy-to-collect risk factors. Dataset was health checkup information collected through a physical examination in a hospital in Beijing. Results showed that the RF model outperformed the other three machine learning methods, and it performed an AUC of 0.92, an accuracy of 0.82, a sensitivity of 0.83, and a specificity of 0.81. The results revealed that the RF model could distinguish high-risk and low-risk populations of hypertension based on easy-to-collect variables. Thus, the RF model has a great application value in the prevention and management of hypertension.

## Data Availability Statement

The raw data supporting the conclusions of this article will be made available by the authors, without undue reservation.

## Ethics Statement

The studies involving human participants were reviewed and approved by the Ethics Committee of the Hefei Institute of Physical Science, Chinese Academy of Sciences. The patients/participants provided their written informed consent to participate in this study. Written informed consent was obtained from the individual(s) for the publication of any potentially identifiable images or data included in this article.

## Author Contributions

HZ and ZM: conceptualization. LG and ZM: funding acquisition. HZ and YX: investigation. XZ and WW: resources. YS: supervision. HZ: Writing – original draft. ZM: Writing – review & editing. All authors contributed to the article and approved the submitted version.

## Funding

This work was supported by the major special project of Anhui Science and Technology Department under Grant 18030801133, and Science and Technology Service Network Initiative under Grant KFJ-STS-ZDTP-079.

## Conflict of Interest

The authors declare that the research was conducted in the absence of any commercial or financial relationships that could be construed as a potential conflict of interest.

## Publisher's Note

All claims expressed in this article are solely those of the authors and do not necessarily represent those of their affiliated organizations, or those of the publisher, the editors and the reviewers. Any product that may be evaluated in this article, or claim that may be made by its manufacturer, is not guaranteed or endorsed by the publisher.
